# Evaluation of PVP/Au Nanocomposite Fibers as Heterogeneous Catalysts in Indole Synthesis

**DOI:** 10.3390/molecules21091218

**Published:** 2016-09-10

**Authors:** Ioanna Savva, Andreas S. Kalogirou, Mariliz Achilleos, Eugenia Vasile, Panayiotis A. Koutentis, Theodora Krasia-Christoforou

**Affiliations:** 1 Department of Mechanical and Manufacturing Engineering, University of Cyprus, P. O. Box 20537, 1678 Nicosia, Cyprus; achilleos.mariliz@ucy.ac.cy; 2 Department of Chemistry, University of Cyprus, P. O. Box 20537, 1678 Nicosia, Cyprus; akalog@ucy.ac.cy (A.S.K.); koutenti@ucy.ac.cy (P.A.K.); 3 Department of Physics, Politehnica University of Bucharest, 313 Splaiul Independentei, Bucharest 060042, Romania; eugenia.vasile27@gmail.com

**Keywords:** electrospinning, polymers, catalysis, indole, gold nanoparticles

## Abstract

Electrospun nanocomposite fibers consisting of crosslinked polyvinylpyrrolidone (PVP) chains and gold nanoparticles (Au NPs) were fabricated, starting from highly stable PVP/Au NP colloidal solutions with different NP loadings, followed by thermal treatment. Information on the morphological characteristics of the fibers and of the embedded Au NPs was obtained by electron microscopy. Cylindrical, bead-free fibers were visualized by Scanning Electron Microscopy (SEM) while Transmission Electron Microscopy (TEM) and Energy Diffraction X-ray (EDX) analysis supported the presence of Au NPs within the fibers and gave information on their morphologies and average diameters. These materials were briefly evaluated as heterogeneous catalytic supports for the gold-catalyzed intramolecular cyclisation of 2‑(phenylethynyl)aniline to form 2-phenyl-1*H*-indole. The performance of the gold catalyst was strongly dependent on the Au NP size, with the system containing the smallest Au NPs being the more effective. Moreover, a slight drop of their catalytic efficiency was observed after three consecutive reaction runs, which was attributed to morphological changes as a consequence of fiber merging.

## 1. Introduction

Significant advancements in the field of nanotechnology in terms of synthesis and materials characterization and processing, have led to new nanostructured materials with enhanced catalytic performance. The combination of inorganic catalytic nanoparticles with polymeric materials has led to cost-effective polymer-based nanocomposites that exhibit superior catalytic activity and selectivity in both homo- and heterogeneous catalysis, including photocatalysis, electrocatalysis and catalytic procedures adopted in organic synthesis [1].

In 1989, Haruta et al. [2], reported the high catalytic activity of active oxide-supported gold nanoparticles (Au NPs) in the low temperature oxidation of CO and H_2_, NO reduction and CO_2_ hydrogenation processes. Since then, nano-sized Au NPs have attracted significant interest in catalytic industrial and environmental processes [3,4].

There are several examples of Au NP-containing colloidal systems that were prepared and employed in homogeneous catalysis; however, these could not be readily recovered from the reaction products [5]. Moreover, nanoparticle agglomeration during the catalytic process can reduce their catalytic activity [6].

To overcome these problems, catalytic Au NPs have been immobilized onto high surface-area insoluble matrices including hydrogels [7,8,9,10], ceramic porous substrates [2], thin films [11,12,13] and fibers [14,15,16].

Recently, electrospinning has evolved into a versatile and powerful technique for the production of polymeric, carbon-based, ceramic and composite nano- and microfibers [17,18,19]. The facile incorporation of catalytic inorganic nanoparticles within electrospun fibers, their high surface areas and porosity and the possibility for further surface functionalization including chemical and physical post-modification approaches, are some of the advantages of electrospun (nano)fibers that render them highly appropriate for catalytic applications.

Although several reports deal with the production of inorganic electrospun fibers employed as insoluble matrices for hosting Au NPs [20,21,22,23], only a limited number of these appear on the fabrication of Au NP-containing polymer-based electrospun nanocomposite fibers. Furthermore, most of these studies have focused on the synthetic and characterization aims [24,25,26,27,28,29,30,31,32], while in terms of applications, the focus has been towards optoelectronics [26,33,34,35,36,37] and sensing [38,39,40,41,42]. To the best of our knowledge, there are only two reports discussing the catalytic performance of Au NP-immobilized polymer nanofibers in organic synthesis [43,44].

To obtain electrospun polymer fibrous mats exhibiting structural and compositional stability in organic or aqueous solvents thus enabling their use as heterogeneous catalytic supports in organic synthesis, fiber crosslinking is required, which usually involves chemical treatment of the fibers [45,46,47,48,49,50]. In this regard, the use of polyvinylpyrrolidone (PVP) is advantageous since insoluble PVP-based fibers can be obtained via thermal treatment of the as-prepared fibrous mats under relatively mild heating conditions [51,52]. In addition, the introduction of specific metal binding functionalities (such as carboxylates and amines) via chemical modification steps can be avoided, since the polymer itself acts as a highly effective steric stabilizer for a wide variety of catalytic metal nanoparticles including Au, Pt, Rh, Ag, Pd, and Cu etc [53,54,55].

Very recently, we fabricated PVP-based electrospun fibrous nanocomposite membranes with embedded Pd and Cu_2_O NPs, that were successfully evaluated as heterogeneous catalytic supports in Heck, Suzuki and click chemistry reactions [52]. Giving further credence to this work, we report below the catalytic efficacy of Au NP-containing PVP-crosslinked electrospun fibers in a typical gold-catalyzed intramolecular cyclisation of 2‑(phenylethynyl)aniline (**1**) to form 2-phenyl-1*H*-indole (**2**).

The gold activation of alkynes towards intramolecular cyclisation has been studied thoroughly and occurs mainly with homogeneous gold complexes [56]. Recently, Au NPs have appeared as suitable heterogeneous catalysts for various organic reactions such as oxidations, reductions, cyclisations and other reactions [57]. Since the intramolecular cyclisation to form indoles with Au NP catalysts has been reported [58] and due to the biological and pharmaceutical importance of indoles [59,60], we chose this as a representative reaction to test the catalytic activity of our PVP/Au nanocomposite fibrous membranes. Indoles are important heterocycles owing to their biological activities and play a prominent role in classic organic synthesis and modern catalysis.

## 2. Results and Discussion

### 2.1. PVP/Au Colloidal Nanohybrids

**Figure 1:**
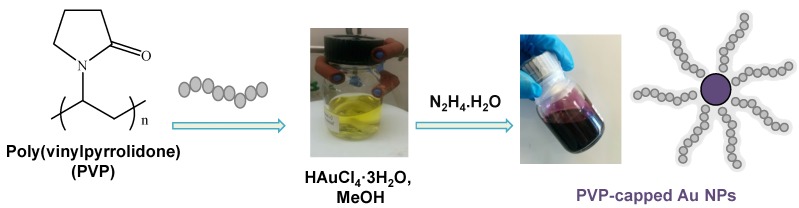
Schematic presentation of the synthetic pathway followed for the generation of highly stable polyvinylpyrrolidone (PVP)/Au colloidal nanohybrid solutions prepared in MeOH and corresponding photographs.

The PVP/Au nanohybrids were prepared in the form of highly stable colloidal solutions in methanol. The reaction process involved the reduction of the Au(III) ions into metallic Au(0) nanoparticles (NPs) upon introducing hydrazine monohydrate as a reducing agent (Figure 1).

**Figure 2:**
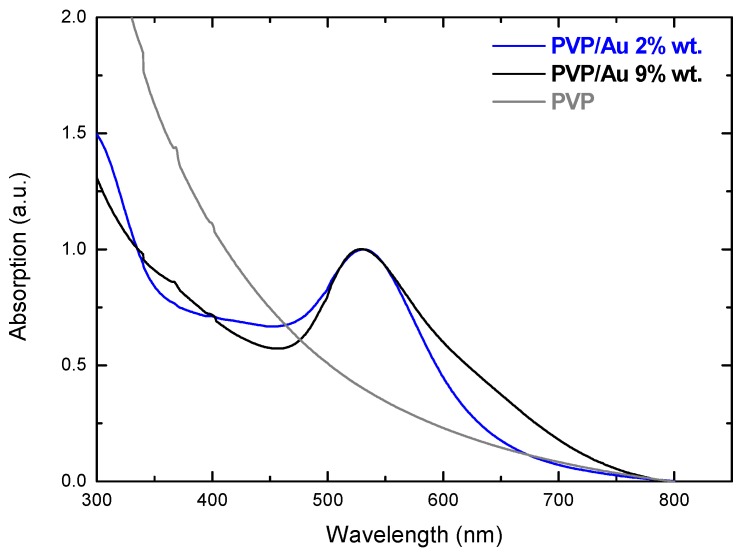
UV-vis spectra of the pristine PVP and PVP/Au colloidal solutions prepared in MeOH, with 2% and 9% wt. Au nanoparticles (NP) loading.

The optical characterization of the PVP and the PVP/Au methanol solutions was performed by UV-vis spectroscopy (Figure 2).

As seen in the spectra of the PVP/Au systems, a characteristic absorption band appeared at around 520 nm, which corresponded to the surface plasmon resonance (SPR) of the Au NPs [61,62]. Spectrum broadening was observed in the case of the system exhibiting the highest Au NP loading (i.e., 9% wt). According to Seong et al. [63], such broadening phenomena can be attributed to electric dipole–dipole interactions and coupling occurring between the plasmons of neighboring particles in cases where nanoparticle agglomeration phenomena occur.

### 2.2. Membrane Fabrication

**Figure 3:**
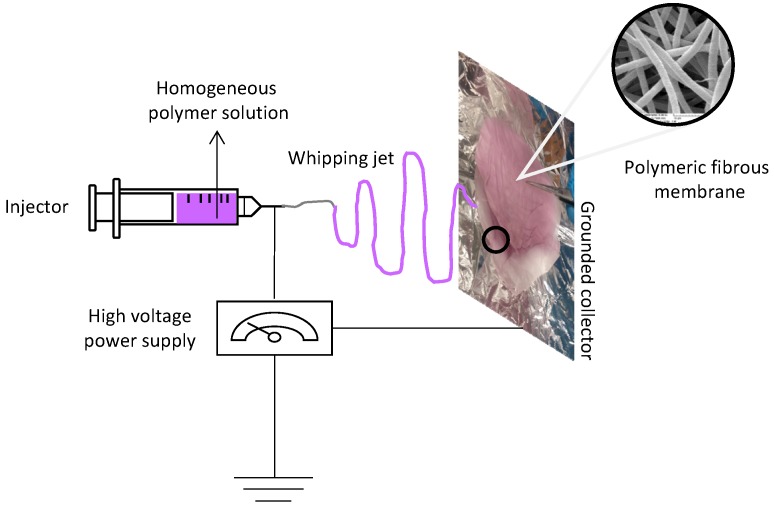
Schematic presentation of the fabrication of polymer-Au NP electrospun fibers starting from highly stable PVP/Au colloidal nanohybrid solutions. Photographs show the nanohybrid PVP/Au solution precursor and the corresponding electrospun PVP/Au nanocomposite membrane.

The PVP/Au NPs solutions were used as precursors for the fabrication of Au NP-containing nanocomposite fibrous mats based on PVP, by means of the electrospinning technique (Figure 3).

Systematic parametric studies helped determine the optimum experimental conditions for the production of fibrous, bead-free PVP/Au nanocomposite fibers. Variable parameters included the polymer solution concentration, the applied voltage, the delivery rate of the solution, the diameter of the needle and the needle-to-collector distance. In Table 1 the optimum electrospinning parameters used for the production of PVP and PVP/Au electrospun fibrous mats with different Au loading (2% and 9% wt), are provided in entries 1, 2 and 3, respectively. With regards to the solution concentration, 10% *w*/*v* was the optimum for the fabrication of continuous, cylindrical fibers without beads, which agreed with our earlier work [64].

**Figure 4:**
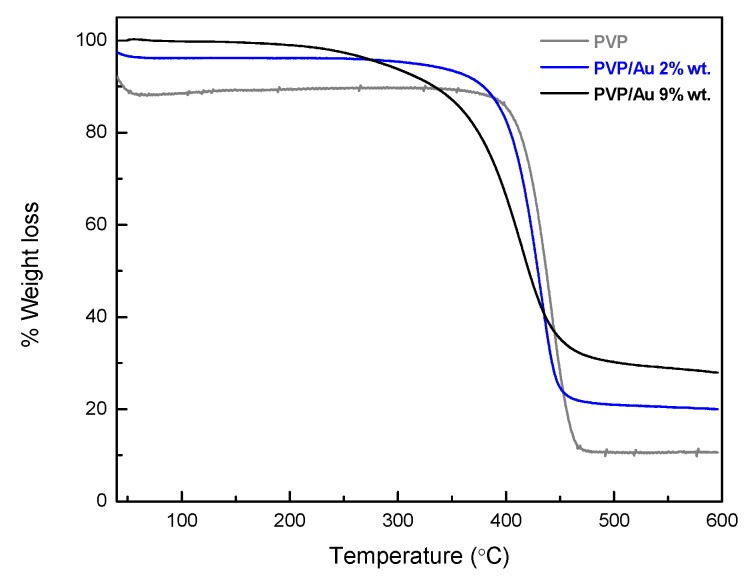
Thermal Gravimetric Analysis (TGA) thermograms of the as-prepared PVP and PVP/Au electrospun fibrous mats with different Au NP loading.

The thermal stability of the as-prepared PVP electrospun fibrous membranes was investigated in the presence and absence of Au NPs by means of thermal gravimetric analysis (TGA). Figure 4 provides the TGA thermograms of the pristine PVP membrane and the nanocomposite PVP/Au systems. As seen in Figure 4, PVP degraded at ~400 °C, in line with our previously reported findings [64]. However, residue formation owing to incomplete combustion at this temperature range was observed, even in the case of the pure PVP system, which prevented the accurate determination of the Au NP content in the PVP/Au systems. 

**Figure 5:**
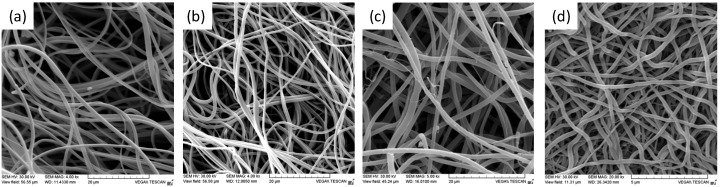
SEM images of the as-prepared pristine PVP (**a**) and of the PVP (**b**), PVP/Au, (2% wt) (**c**) PVP/Au, (9% wt) (**d**) electrospun fibrous mats obtained upon crosslinking.

Thermal crosslinking of the fibrous mats was carried out to prevent membrane dissolution and consequently NP leaching of the heterogeneous catalytic supports in the organic reaction media. As demonstrated in Figure 5 and in line with our earlier work on the fabrication of Pd- and Cu_2_O-containing electrospun heterogeneous catalysts [52], the thermal crosslinking step succeeded without altering the morphology of the fibers and effectively prevented the leaching of the catalytic metal nanoparticles from the polymer-based substrate during the catalytic reaction process.

SEM images of the as-prepared (non-crosslinked) PVP membrane (Figure 5a) and of the thermally crosslinked PVP and PVP/Au electrospun nanocomposite membranes (Figure 5b–d) indicated in all cases continuous, bead-free cylindrical fibers, under the optimum electrospinning conditions employed. Furthermore, no significant changes were observed in the fiber morphology in the presence of the embedded Au NPs, compared to the pristine PVP fibers. Finally, the morphological characteristics of the fibers, i.e., fiber continuity and cylindrical morphology, were not significantly altered upon increasing the Au NP loading % cf. Figure 5c vs. Figure 5d.

**Figure 6:**
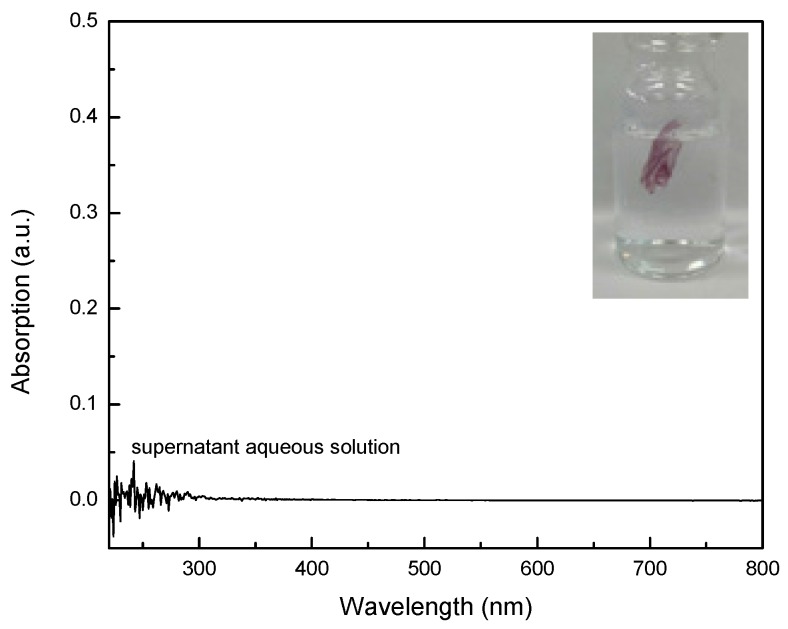
UV-vis spectrum of the supernatant aqueous solution recorded after immersing the crosslinked PVP/Au electrospun fibrous mat in water at ambient temperature for several days.

To verify the success of the crosslinking process, the thermally-treated membranes were immersed in water at ambient temperature for several days and subsequently, the UV-vis spectrum of the supernatant was recorded. As seen in Figure 6, no absorption signals corresponding to either PVP or Au NPs were observed. The absence of leaching phenomena was further supported by the photograph of the crosslinked PVP/Au electrospun fibrous mat immersed in water provided in the same figure.

**Figure 7:**
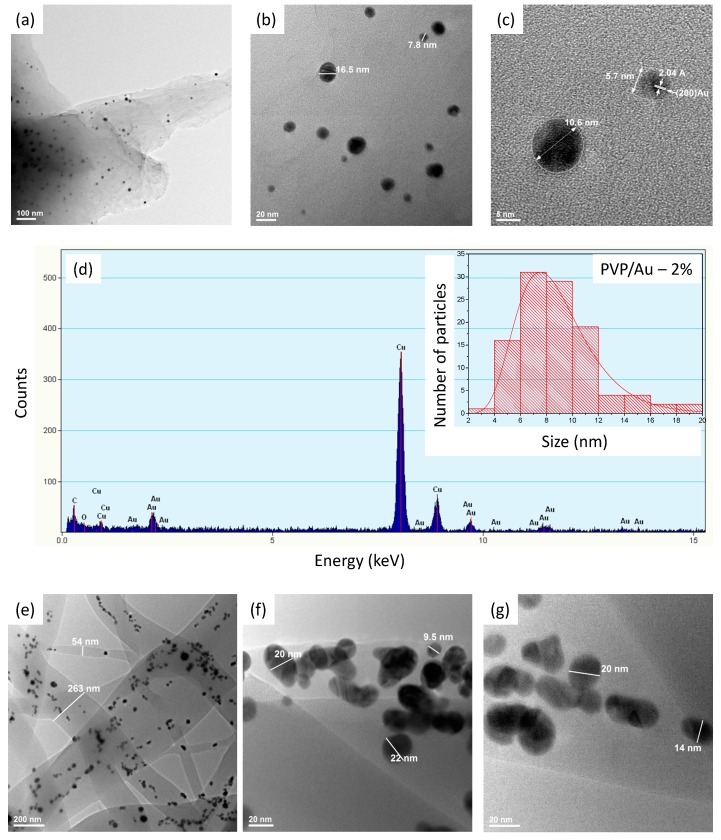

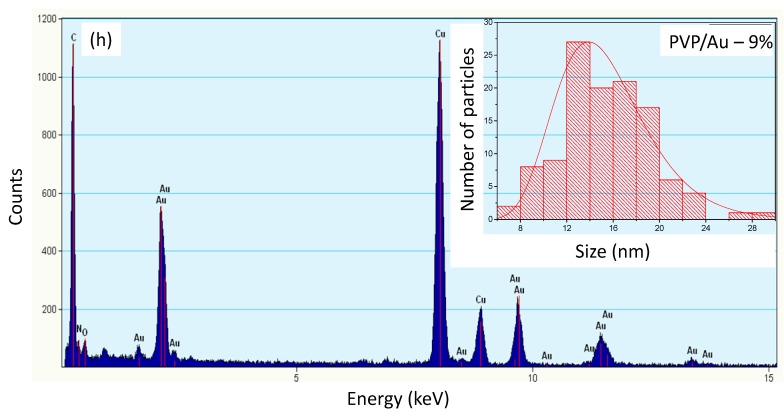
Transmission Electron Microscopy (TEM) bright field images (**a**–**c**), average Au NP size distribution histogram and Energy Diffraction X-ray (EDX) (**d**) corresponding to the PVP/Au—2% wt system. TEM bright field images (**e**–**g**), average Au NP size distribution histogram and EDX (**h**) corresponding to the PVP/Au—9% wt system.

The resulting materials were also visualized by TEM to obtain information on the size and morphological characteristics of the Au NPs embedded within the polymer fibers. Figure 7 depicts the corresponding TEM micrographs of the crosslinked fibrous materials with a 2% and 9% wt Au NP content. The corresponding EDX spectra and characteristic NP size distribution histograms are also provided in the same figure. EDX analysis confirmed the existence of Au NPs within the fibers. The presence of Cu is attributed to the Cu grid employed in TEM investigations. As seen in the images and in line with the size distribution profiles corresponding to the two PVP/Au systems, the PVP/Au (2% wt) system is characterized by smaller average NP diameters (8.86 ± 3.04 nm) compared to the PVP/Au (9% wt) analogue exhibiting average NP diameters of 15.40 ± 3.93 nm. According to Haruta et al. [65], Au NPs with sizes below 20 nm, employed in low-temperature CO oxidation, are exceedingly good catalysts, whereas in contrast, Au NPs of diameter above 20 nm were devoid of significant catalytic activity. Moreover, there is an exponential inverse relationship between the particle size and the catalytic activity. In view of this, there is continued interest in reducing particle size [4]. 

### 2.3. Catalysis

**Scheme 1:**
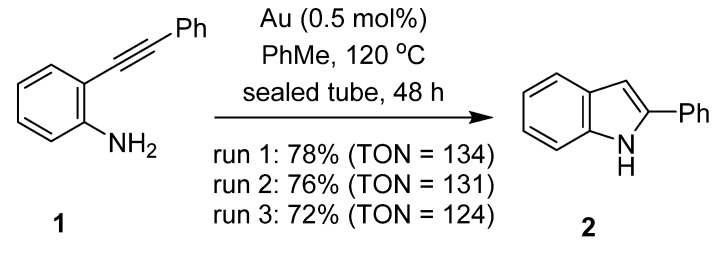
Cyclisation of 2-(phenylethynyl)aniline (**1**) to 2-phenyl-1*H*-indole (**2**).

**Figure 8:**
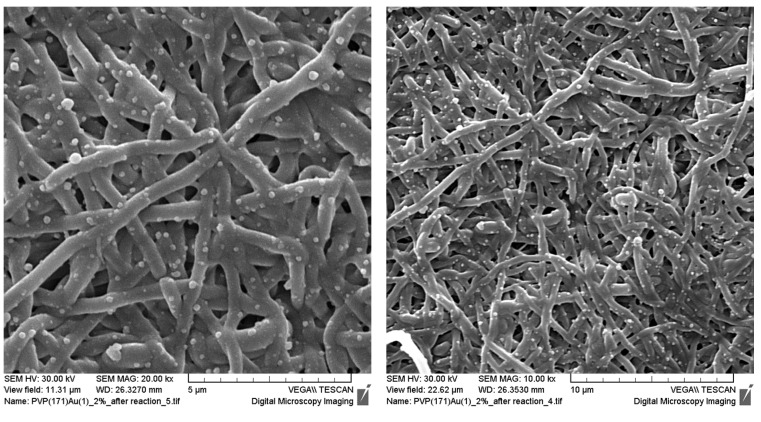
SEM images of the PVP/Au (2% wt) crosslinked membrane recovered after three reaction runs.

Upon preparation of the polymer-supported Au NPs, a brief investigation of their catalytic activity in classical gold catalysis was pursued. Having in hand two catalytic systems with different loadings of gold (0.0525 and 0.272 mmol Au/g of catalyst corresponding to 2% wt and 9% wt, respectively), we investigated the gold-catalyzed intramolecular cyclisation of 2-(phenylethynyl)aniline (**1**). To compare our catalyst to the literature we used similar reaction conditions to those of Perea-Buceta et al. [58], who used carbon-supported Au NPs to perform the same reaction. When our catalyst with the lower loading was used (2% wt corresponding to 0.5 mol % Au), under heating in PhMe at ca. 120 °C (sealed tube), complete consumption of the starting material was observed after 48 h and isolation of 2-phenyl-1*H*-indole (**2**) in 78% yield (Scheme 1). For comparison, the carbon-supported Au NPs in the literature reaction gave a yield of 87% after 24 h at 90 °C in the presence of a lower loading of 0.24 mol % Au. Our catalyst was reused twice to give 76% and 72% yields of indole **2**, respectively. The slight drop in activity after three consecutive reaction runs was attributed to morphological changes of the fibers as a consequence of fiber merging, as observed from the SEM images of the fibers before and after three catalytic reaction runs (Figure 8). This result was in line with our previous work with PVP membranes when similar structural changes were observed [52].

Interestingly, when our PVP-supported gold catalyst with a higher loading of Au (9% wt corresponding to 0.5 mol% Au) was used to perform the same reaction, the consumption of aniline **1** was incomplete even after 7 days when the reaction was stopped and chromatographed to give product **2** in 60% yield and recovered aniline **1** (24%). Tentatively, this indicated that higher gold loadings led to larger particle size and, therefore, a reduction of the surface area of the metal available for catalysis. The results were in agreement with the TEM investigations, which confirmed the particle size differences between the two systems: the one with the highest NP loading displayed larger particle diameters compared to the lowest NP loading analogue. Considering the exponential inverse relationship between the Au NP size and the catalytic activity, our findings on the catalytic efficacy differences existing between the two systems can be rationalized based on NP size differences. 

## 3. Materials and Methods

### 3.1. Solvents and Reagents

Polyvinylpyrrolidone (PVP, average molecular weight = 1,300,000), gold(III) chloride trihydrate (HAuCl_4_·3H_2_O > 99.9%), and hydrazine monohydrate (N_2_H_4_·H_2_O) were purchased from Sigma-Aldrich (St. Louis, MO, USA). Methanol (Analytical grade, ACS reagent) was purchased from Scharlau (Barcelona, Spain). The above-mentioned reagents were used as provided by the supplier without further purification. Toluene was dried by azeotropic removal of water using a Dean-Stark apparatus (SciLabware Ltd, Stoke-on-Trent, UK). All volatiles were removed under reduced pressure. All reaction mixtures and column eluents were monitored by TLC using commercial glass backed thin layer chromatography (TLC) plates (Merck Kieselgel 60 F_254_, (Merck KGaA, Darmstadt, Germany)). The plates were observed under UV light at 254 and 365 nm. The technique of dry flash chromatography was used throughout for all non-TLC scale chromatographic separations using Merck Silica Gel 60 (less than 0.063 mm) (Merck KGaA, Darmstadt, Germany) [66]. 2-(Phenylethynyl)aniline (**1**) was prepared according to the literature [67].

### 3.2. Synthesis of PVP/Au Colloidal Nanohybrids

The PVP/Au nanohybrid colloidal systems (solution concentration: 10% *w/v* with respect to the polymer mass), with different Au % loading, (i.e., mols vinyl pyridine units/mols gold salt = 171:1 and 31:1 corresponding to 2% and 9% wt loading percentage, respectively) were prepared as follows: In the case of the system corresponding to the 171:1 molar ratio, in a round-bottomed flask (50 mL), PVP (1.076 g, 8.38 × 10^−4^ mmol, 9.8 mmol per VP unit) was dissolved in dry MeOH (8 mL) and was stirred at room temperature. To the solution, HAuCl_4_·3H_2_O (22.5 mg, 0.0571 mmol, 0.0525 mmol Au/g) dissolved in dry MeOH (2 mL) was added with the aid of the syringe. The mixture was left to stir at room temperature until complete solubilization of the HAuCl_4_·3H_2_O salt. Subsequently, hydrazine monohydrate (27.7 μL, 28.6 mg, 0.571 mmol) was added using a micropipette. The yellow solution turned immediately dark purple and it was left to stir under ambient conditions for another 2 h. The final polymer-Au nanohybrid solution was highly stable and no agglomeration or destabilization phenomena were observed even after several months.

### 3.3. Fabrication of Electrospun PVP/Au Fibrous Membranes

The PVP/Au colloidal nanohybrid solution was employed for the fabrication PVP/Au nanocomposite fibrous membranes by means of the electrospinning technique. All electrospinning experiments were performed at ca. 20 °C. Equipment included a controlled-flow, four-channel volumetric microdialysis pump (KD Scientific, Model: 789252), syringes with specially connected spinneret needle electrodes, a high-voltage power source (10–50 kV) and a custom-designed, grounded target collector, inside an interlocked Faraday enclosure safety cabinet. Systematic parametric studies were performed by varying the applied voltage, the needle-to-collector distance, the needle diameter and the flow rate so as to determine the optimum experimental conditions for obtaining bead-free fibrous materials.

### 3.4. Membrane Crosslinking

Insoluble PVP/Au electrospun nanocomposite fibrous mats were obtained via thermal crosslinking of the as prepared PVP/Au electrospun fibers [52]. Thermal crosslinking was realized upon placing the membranes in an oven at 170 °C for 5 h.

### 3.5. Catalysis

Gold catalyzed cyclisation of 2-(phenylethynyl)aniline (**1**) to 2-phenyl-1*H*-indole (**2**): To a stirred solution of 2-(phenylethynyl)aniline (**1**) (38.2 mg, 0.20 mmol) in dry PhMe (1 mL) at ca. 20 °C, was added the PVP/Au electrospun membrane (15.4 mg, 2% wt, 0.5 mol %, 0.0525 mmol Au/g of catalyst). The mixture was heated to ca. 120 °C in a sealed tube until complete consumption of the starting material (TLC, 48 h). The mixture was then cooled to ca. 20 °C and the polymer filtered and washed with *t*-BuOMe (10 mL). The membrane was reused in subsequent reactions without further treatment. The organic washings were combined, adsorbed onto silica and chromatographed (*n*-hexane/DCM, 60:40) to give 2-phenyl-1*H*-indole (**2**) (29.8 mg, 78%) as colorless plates, mp (hotstage) 183–185 °C (from *n*-hexane, lit. 186–187 °C [68]); *R*_f_ 0.50 (*n*-hexane/DCM, 60:40); *ν*_max_/cm^−1^ 3443m (N-H), 3050w (C-H), 1605w, 1541w, 1493w, 1481m, 1456m, 1447m, 1404m, 1352m, 1339m, 1298m, 1242m, 1231m, 1190m, 1115m, 1074w, 1049w, 1028w, 1009w, 932w, 907w, 797m, 762s, 743s; *δ*_H_(500 MHz; CDCl_3_) 10.66 (1H, s, N*H*), 7.86 (2H, d, *J* 7.5 Hz, Ar *H*), 7.57 (1H, d, *J* 7.9 Hz, Ar *H*), 7.45 (2H, dd, *J* 7.7, 7.7 Hz, Ar *H*), 7.44 (1H, d, *J* 8.2 Hz, Ar *H*), 7.31 (1H, dd, *J* 7.4 Hz, Ar *H*), 7,10 (1H, dd, *J* 7.4, 7.4 Hz, Ar *H*), 7.02 (1H, d, *J* 7.5 Hz, Ar *H*), 6.90 (1H, s, Ar *H*); *δ*_C_(125 MHz; CDCl_3_) 138.8 (s), 138.4 (s), 133.6 (s), 130.2 (s), 129.8 (d), 128.3 (d), 125.9 (d), 122.7 (d), 121.1 (d), 120.5 (d), 112.0 (d), 99.9 (d); *m/z* (MALDI-TOF) 193 (M^+^, 90%), 165 (100), identical to that reported [68].

### 3.6. Characterization Methods

The UV-vis spectra of the PVP/Au nanohybrids stabilized in MeOH were recorded on a Jasco V-630 UV-vis spectrophotometer operating at ca. 20 °C, after appropriate dilution of the as prepared colloidal solutions. High Resolution Transmission Electron Microscopy (HRTEM) investigations of the membranes were performed by using a TECNAI F30 G2 S-TWIN microscope operated at 300 kV equipped with energy dispersive X-ray spectrometer (EDX). Samples were placed into a double copper grid (oyster) to be visualized by TEM. The morphological characteristics of the nanocomposite membranes were also determined by scanning electron microscopy (SEM) (Vega TS5136LS-Tescan, Brno, Czech Republic). The samples were gold-sputtered (~15 nm) (sputtering system K575X Turbo Sputter Coater—Emitech, Quorum Technologies Ltd., West-Sussex, UK) prior to SEM inspection. Thermal Gravimetric Analysis (TGA) measurements were performed on a Q500 TA instrument (TA Instruments, New Castle, DE, USA) under argon flow at a heating rate of 10 °C/min.

For the characterisation of the final product obtained from the catalytic reaction: Melting points were determined using a PolyTherm-A, Wagner & Munz, Koefler-Hotstage Microscope apparatus (Wagner & Munz, Munich, Germany). IR spectra were recorded on a Shimadzu FTIR-NIR Prestige-21 spectrometer (Shimadzu, Kyoto, Japan) with Pike Miracle Ge ATR accessory (Pike Miracle, Madison, WI, USA) and strong, medium and weak peaks are represented by s, m and w, respectively. ^1^H and ^13^C-NMR spectra were recorded on a Bruker Avance 500 machine (at 500 and 125 MHz, respectively, (Bruker, Billerica, MA, USA)). Deuterated solvents were used for homonuclear lock and the signals are referenced to the deuterated solvent peaks. CH assignments are made based on DEPT 135 spectroscopy. MALDI-TOF mass spectra were recorded on a Bruker Autoflex III Smartbeam instrument (Bruker).

## 4. Conclusions

Cost-effective, polymer-based heterogeneous catalytic supports comprised of insoluble PVP electrospun fibers and embedded catalytic Au NP were successfully prepared and evaluated in a typical gold-catalyzed intramolecular cyclisation reaction involving the transformation of 2-(phenylethynyl)aniline to 2-phenyl-1*H*-indole. Based on the experimental findings, the catalytic efficiency of these materials depended strongly on the size of the embedded Au NPs. More precisely, the system containing the smallest Au NPs exhibited good catalytic efficiency, i.e., complete consumption of the starting material and product isolation at 78% yield, whereas the corresponding material enclosing the largest Au NPs led to an incomplete reaction and a lower product yield.

## Figures and Tables

**Table 1:** Optimum experimental parameters employed for the fabrication of the PVP (pristine) and the PVP/Au nanocomposite electrospun fibrous membranes.

Electrospinning Conditions					
Entry	Au Loading (% wt)	Needle (G)	Voltage (KV)	Needle-to-Collector Distance (cm)	Flow Rate (mL/h)
1	0	16	25.0	28	1.8
2	2	16	22.5	20	2.0
3	9	16	22.5	15	1.0
